# Effect of Beta 3 Adrenoreceptor Modulation on Patency of the Ductus Arteriosus

**DOI:** 10.3390/cells9122625

**Published:** 2020-12-07

**Authors:** Alessandro Pini, Camilla Fazi, Patrizia Nardini, Maura Calvani, Sergio Fabbri, Alessandro Guerrini, Giulia Forni, Giancarlo La Marca, Arianna Carolina Rosa, Luca Filippi

**Affiliations:** 1 Department of Experimental and Clinical Medicine, University of Florence, 50139 Florence, Italy; patrizia.nardini@unifi.it; 2 Department of Health Sciences, University of Florence, 50139 Florence, Italy; camillafazi@hotmail.it; 3 Department of Paediatric Haematology-Oncology, A. Meyer University Children’s Hospital, 50139 Florence, Italy; maura.calvani@meyer.it; 4 Department of Neuroscience, Psychology, Drug Research and Child Health, Section of Pharmacology and Toxicology, University of Florence, 50139 Florence, Italy; sergio.fabbri@unifi.it; 5 Department of Veterinary Medical Sciences, University of Bologna, 40064 Ozzano dell’Emilia, Italy; alessandro.guerrini5@unibo.it; 6 Department of Neuroscience, Psychology, Drug Research and Child Health, Section of Pediatric Neurosciences, “A. Meyer” University Children’s Hospital, 50139 Florence, Italy; giuliaforni1984@libero.it (G.F.); giancarlo.lamarca@meyer.it (G.L.M.); 7 Department of Scienza e Tecnologia del Farmaco, University of Turin, 10125 Turin, Italy; ariannacarolina.rosa@unito.it; 8 Division of Neonatology and NICU, Department of Clinical and Experimental Medicine, University of Pisa, 56126 Pisa, Italy

**Keywords:** β3-adrenorceptor, ductus arteriosus, cancer, pregnancy

## Abstract

β3-adrenoreceptor (β3-AR), a G-protein coupled receptor, has peculiar regulatory properties in response to oxygen and widespread localization. β3-AR is expressed in the most frequent neoplasms, also occurring in pregnant women, and its blockade reduces tumor growth, indicating β3-AR-blockers as a promising alternative to antineoplastic drugs during pregnancy. However, β3-AR involvement in prenatal morphogenesis and the consequences of its blockade for the fetus remain unknown. In this study, after the demonstrated expression of β3-AR in endothelial and smooth muscle cells of ductus arteriosus (DA), C57BL/6 pregnant mice were acutely treated at 18.5 of gestational day (GD) with indomethacin or with the selective β3-AR antagonist SR59230A, or chronically exposed to SR59230A from 15.5 to 18.5 GD. Six hours after the last treatment, fetuses were collected. Furthermore, newborn mice were treated straight after birth with BRL37344, a β3-AR agonist, and sacrificed after 7 h. SR59230A, at the doses demonstrated effective in reducing cancer progression (10 and 20 mg/kg) in acute and chronic mode, did not induce fetal DA constriction and did not impair the DA ability to close after birth, whereas at the highest dose (40 mg/kg), it was shown to cause DA constriction and preterm-delivery. BRL37344 administered immediately after birth did not alter the physiological DA closure.

## 1. Introduction

β3-adrenoreceptor (β3-AR) belongs to a large family of G-protein coupled receptors, classified in three subfamilies: α1-ARs, α2-ARs, and β-ARs (β1-AR, β2-AR, and β3-AR). Since its discovery in 1989 [1], β3-AR has attracted the interest of the scientific community for its peculiar regulatory properties, its widespread tissue localization, and its involvement in numerous physiopathological processes. The pharmacologic properties of β3-AR reflect the divergence of its molecular structure from other β-AR isoforms. β3-AR differs from both β1- and β2-ARs in the third intracellular loop and in the intracellular C-terminal domain, lacking the sites subjected to adrenoreceptor kinase-mediated regulation and the consensus sequence for protein kinase A [2]. This structure makes β3-AR more resistant to the desensitization compared with other β-AR isoforms [2] and appoint it as an interesting therapeutic target for chronic treatments. β3-AR also showed a particular regulatory mechanism in response to the oxygen stimulus, increasing its expression level and its functional responsiveness upon exposure to hypoxia [3].

Although the early studies documented β3-AR expression only in brown and white adipose tissues, where it promotes thermogenesis and lipolysis, respectively [4,5,6], more recently, its presence was demonstrated in several anatomical districts, such as the heart and blood vessels, central nervous system, gastrointestinal tract, bladder, prostate, and near-term myometrium [4,7,8,9].

In the cardiovascular system, β3-AR activation induces a negative inotropic effect in the myocardium [10] and a vasodilatation in the blood vessels [11,12], acting on vascular smooth muscle cells [7,10]. β3-AR activation was also demonstrated to promote angiogenesis, inducing the proliferation of endothelial cells [13] and increasing VEGF release, especially in hypoxic conditions, through the nitric oxide (NO) pathway [3].

Among others, this latter feature contributed towards making β3-AR an attractive alternative target potentially suitable for cancer treatment. A growing body of evidence indicates that β3-AR contributes to cancer progression and its blockade reduces tumor growth, limiting cell proliferation and vascularization [14,15,16,17,18]. Besides melanoma and neuroblastoma, β3-AR expression has been also documented in haematological [19], colorectal [20], and breast [21] malignancies, which represent the most frequent neoplasms occurring in pregnant women [22]. As the use of antineoplastic drugs during pregnancy is matter of concern because of their teratogenic and abortifacient effects [23], a new therapeutic approach based on β3-AR-blockers could represent a promising alternative. However, considering the relatively hypoxic fetal environment and the peculiar β3-AR up-regulation in response to low oxygen, fetal tissues might be particularly sensitive to β3-AR selective compounds and their potential side effects should be taken into account. To the best of our knowledge, except for scattered data reporting weak umbilical vessel vasodilatation [24] and an increase of fetal adipocyte thermogenesis exerted by β3-AR activation [25], the involvement of this receptor in prenatal morphogenesis and the consequences of its blockade for the fetus remain an almost totally unexplored field. However, the expression of β3-AR by cells of the fetal cardiovascular system makes this latter apparatus a possible target of β3-AR blockers and, hence, an Achilles heel of possible anti-cancer treatments based on these drugs. In particular, the possible unwanted side effects of β3-AR-blockade could involve the ductus arteriosus (DA), which is known to properly work under hypoxic conditions [26,27]. DA is an extracardiac fetal shunt connecting the main pulmonary trunk to the descending aorta in order to divert most of the right ventricular blood output away from the collapsed lungs to the systemic circulation. During fetal life, its patency is maintained by low partial pressure of oxygen (pO_2_), as well as by high levels of the endogenous vasodilators prostaglandin E2 (PGE2) and NO [27,28,29]. After delivery, the rapid increase in pO_2_ and decrease in these vasodilator autacoids induces a prompt DA closure within 24–72 h [26,30]. On the other hand, pharmacologically induced prenatal constriction of DA can result in severe cardiac dysfunction and lung hypertension, potentially fatal to the fetus. In this in vivo study, we investigated the effect of SR59230A, a β3-AR selective antagonist, on DA of fetal and newborn mice.

## 2. Materials and Methods

### 2.1. Animals

C57BL/6 mice (Charles Rivers, Wilmington, MA, USA) at gestation day (GD) 15.5 were housed one per cage in a limited-access animal facility under controlled environment conditions (room temperature at 22 °C and 12-h light/12-h dark cycle) and maintained on commercial solid food and tap water available ad libitum. The general conditions and bodyweight of the mice were daily assessed and registered. The experimental protocol was designed according to the European Union (EU) guidelines for animal care procedures and the Italian legislation (DLgs 26/2014) application of the EU Directive 2010/63/EU. The animal study was reviewed and approved by Research permit 366/2019-PR approved by the Italian Ministry of Health.

### 2.2. Maternal Administration

**Figure 1:**
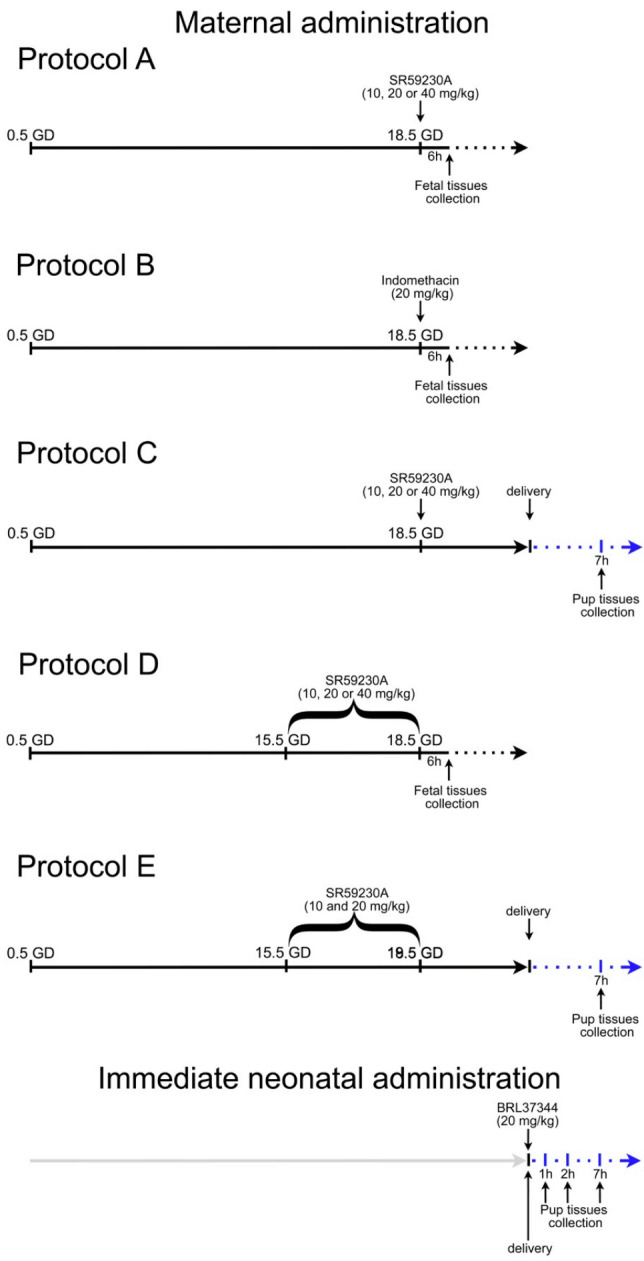
Drug treatment protocols. Maternal administration: seven dams per group were treated at the indicated gestation days (GD) (day 0.5 = presence of vaginal plug). Protocols A and B studied the effects of a single dose of SR59230A or indomethacin respectively on 18.5 GD on fetal ductus arteriosus (DA) (tissues harvested 6 h after treatments); protocol C studied the effects of a single dose of SR59230A on 18.5 GD on newborn DA (tissues harvested 7 h after delivery); protocol D studied the effects of maternal prolonged SR59230A exposure (daily treatment from GD 15.5 to 18.5) on fetal DA (tissues harvested 6 h after the last treatment); protocol E studied the effects of prolonged maternal exposure to SR59230A (daily treatment from GD 15.5 to 18.5) on newborn DA (tissues harvested 7 h after delivery). Immediate neonatal administration: newborn mice were injected with BRL37344 within 2 min after birth and tissues were harvested after 1 h, 2 h and 7 h.

For studying the effect of the β3-AR antagonist in prenatal DA patency, dams were randomly divided in 6 experimental groups of 7 animals each, according to a simple randomization design. The selective β3-AR antagonist SR59230A (Sigma Aldrich, St. Louis, MO, USA) was reconstituted in physiological solution and intraperitoneally administered at different time points during mid and late gestation according to the treatment protocol (Figure 1). Indomethacin, an inhibitor of cyclo-oxygenase (COX) enzyme, was administrated by oral gavage on 18.5 GD. Untreated animals were used as controls (CTRL).

In protocol A, the dams were treated on 18.5 GD with a single dose of SR59230A at 10, 20 or 40 mg/kg (acute treatment).

In protocol B, the dams received a single dose of 20 mg/kg indomethacin on 18.5 GD (acute treatment). Caesarian deliveries were carried out at 6 h after the last drug administration and fetal tissues were immediately harvest for morphological and biochemical analysis.

In protocol C, the dams received the same treatments as protocol A, but pregnancies were allowed to continue until term gestation, which varied from 19.5 to 20.5 GD. Tissues were harvested from newborn pups 7 h after delivery (acute treatment).

In protocol D, pregnant females were daily treated with SR59230A at 10, 20 or 40 mg/kg from day 15.5 to 18.5 of gestation (total pregnancy period 18.5 days). Caesarian deliveries were carried out 6 h after the last drug administration and fetal tissues were immediately harvested for morphological and biochemical analysis (chronic treatment).

In protocol E, pregnant females received the same treatments of the protocol D, but pregnancies were allowed to continue until term gestation, which varied from 19.5 to 20.5 GD. The tissues were harvested from newborn pups 7 h after delivery (chronic treatment).

### 2.3. Immediate Neonatal Administration

To exclude a possible involvement of β3-AR in postnatal DA closure, newborn mice were subcutaneously injected with BRL37344, a specific β3-AR agonist, 20 mg/kg, within 2 min after birth and the tissues were harvested after 1, 2, and 7 h after treatment.

### 2.4. Measurement of SR59230A Blood Concentration

SR59230A blood concentration was measured on dried blood spot (DBS) by a liquid-chromatography tandem-mass spectrometry (TMS) assay in the pregnant mice at stage GD 18.5 before caesarian section and in the fetuses. TMS assay was conducted according to the published method [31]. Briefly, analysis was performed using a AB SCIEX 5500 QTRAP 5500 mass spectrometer (McKinley scientific, Sparta, NJ, USA) coupled to an Agilent 1260 Infinity HPLC capillary system (Agilent Technologies, Santa Clara, CA, USA). Chromatographic separation was achieved by a Gemini C6-Phenyl column, 3 µm, 100 × 2 mm (Phenomenex, Torrance, CA, USA), operating in gradient mode. For SR59230A extraction each DBS sample was added with 200 µL of methanol and 0.1% of formic and incubated at 37 °C in an orbital shaker for 25 min. Unknown concentration of sample was calculated using a calibration curve obtained by spiking whole blood from untreated mice with increasing drug concentrations. The measured values are reported as mean ± SD and are representative of three independent experiments. To facilitate comparison, fetal SR59230A blood concentration are also expressed as percentage of those measured in the mother.

### 2.5. Real Time RT-PCR

DA from fetuses and bladder (taken as positive control tissue) from dams were rapidly excised and used to evaluate β3-AR expression (Adrb3), using Phosphoglycerate Kinase 1 (Pgk1) as a normalizer gene. The tissues were homogenized, using a TissueLyserII (Qiagen^®^, Hilden, Germany) and RNA extraction was carried out using QIAzol Lysis Reagent following the manufacturer’s protocol (Qiagen^®^). After an electrophoretic run on a 0.8% agarose gel to verify RNAs integrity, the extracted RNAs were then reverse-transcribed with QuantiTect Reverse Transcription Kit (Qiagen^®^). The cDNAs obtained were verified by an end-point PCR with primers of an housekeeping gene (β-Actin: forw 5′-tctttgcagctccttcgttg-3′; rev 5′-gatgctccccgggctgtatt-3′) drawn between exons 1-2, and finally amplified by real time RT-PCR using a Taqman TM Fast Advanced Master Mix (Thermo Fisher Scientific, Waltham, MA, USA). Primers and probes for comparative RT-PCR (Rotor-Gene Q Software, Qiagen^®^, Hilden, Germany), with bladder as a calibrator tissue, were included with the following reference codes: Pgk1 (Thermo Fisher Scientific Mm00435617_m1), Adrb3 (Thermo Fisher Scientific Mm00442669_m1). The RT-PCRs were conducted in triplicate according to the manufacturer’s protocol (Taqman TM Fast Advanced Master Mix, Thermo Fisher Scientific, Waltham, MA, USA).

### 2.6. Flow Cytometry Analysis

DA were mechanically dissociated, and single cells suspended in staining buffer. Cells were then treated with FcR blocking reagent and stained with primary antibodies (see Table 1) followed by appropriate fluorochrome-conjugated secondary antibodies. After staining, cells were subjected to flow cytometry by using a Miltenyi Biotec MACSQuant Analyzer 10 (Miltenyi Biotec, Bergisch Gladbach, Germany). Results were analyzed by using FlowlogicTM Software (Miltenyi Biotec).

### 2.7. Histological Staining and Morphometrical Analysis

Fetuses or pups obtained from each experimental group were decapitated, abdominally opened and fixed in 4% paraformaldehyde in 0.1 M phosphate buffered saline (PBS) pH 7.4 for 1 h. The whole thorax was paraffin embedded and cut in frontal or transverse planes. Serial sections (6 μm thick) of DA were used for immunofluorescence, hematoxylin/eosin, Mallory’s trichrome (Bioptica, Milan, Italy), and paraldehyde fuchsin staining. Prior to use, the sections were deparaffinized and rehydrated using xylene and graded ethanol series. For immunofluorescence, rehydrated sections were treated with antigen retrieval solution (citrate buffer, 10mM, pH 6; Sigma, St Louis, MO, USA) at 100 °C for 10 min, cooled at room temperature (RT) for 30 min, washed in PBS, and blocked with 1.5% bovine serum albumin (BSA, Applichem, Darmastad, Germany) in PBS for 20 min at room temperature (RT) to minimize non-specific binding. Sections were then incubated overnight at 4 °C with primary antibodies diluted in BSA 1.5% PBS (see Table 1) and double labelling were performed as follows: β3-AR/α-SMA, β3/CD31. The day after, the sections were thoroughly washed in PBS and incubated for 2 h at RT in the dark with appropriate fluorochrome-conjugated secondary antibodies diluted in BSA 1.5% PBS (see Table 1). Subsequently, the specimens were rinsed three times with PBS and mounted in aqueous medium (Fluoroshield^™^ with DAPI, Thermo Fisher Scientific). The immunolabeled sections were observed under an epi-fluorescence Olympus BX40 microscope coupled to analySIS∧B Imaging Software (Olympus, Milan, Italy) equipped with 20× and 40× objectives.

### 2.8. Determination of Vessel Caliber

Serial sections of fetal and newborn mice were used for quantitative analysis of DA diameter normalized to that of the corresponding transverse aortic arc (AO) [32]. Briefly, DA and aorta inner diameters were measured with a microscope (Nikon, Tokyo, Japan) fitted with a micrometer eyepiece. The diameter of DA lumen was determined at its narrowest point. Measurements were done by a trained histologist blinded to treatment group (AP) and expressed as a DA/AO ratio. At least 20 fetuses of 5 litters were evaluated for each experimental group.

### 2.9. Statistical Analysis

Data are expressed as mean ± S.E.M. Statistical analysis was performed by one-way analysis of variance (ANOVA) test followed by Tukey comparison test. Calculations were carried out using a GraphPad Prism 6.0 statistical program (GraphPad Software, San Diego, CA, USA) and *p* < 0.05 was considered significant.

## 3. Results

### 3.1. SR59230A Blood Concentration

To verify that SR59230A was able to cross the placental barrier its blood concentration was measured both in dams at stage GD 18.5 immediately before caesarian section, and in fetuses of each experimental group (Table 2). TMS assay demonstrated that the fetal blood contained 68.66 ± 2.47% of the SR59230A present in maternal blood, confirming that SR59230A can cross the placental barrier.

### 3.2. β3-ARs Expression on DA

**Figure 2:**
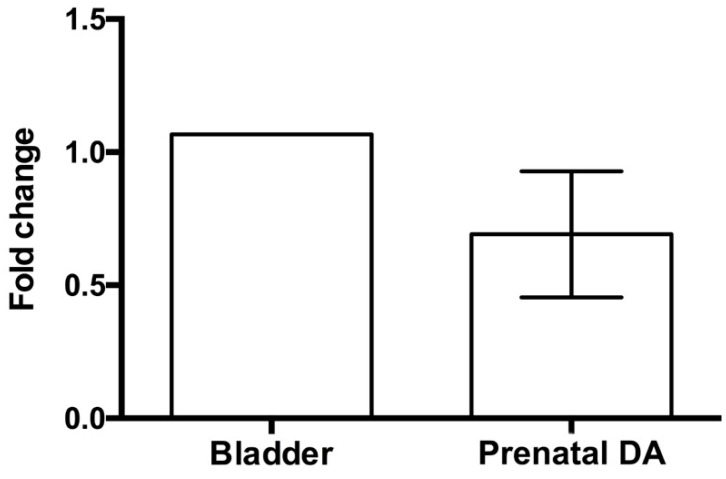
Expression of β3-AR mRNA on ductus arteriosus (DA) of 18.5 mouse fetuses. The results are represented as fold change in DA versus bladder, assumed as positive control. Data are the mean ± SEM of 5 independent experiments, each conducted in triplicate.

**Figure 3:**
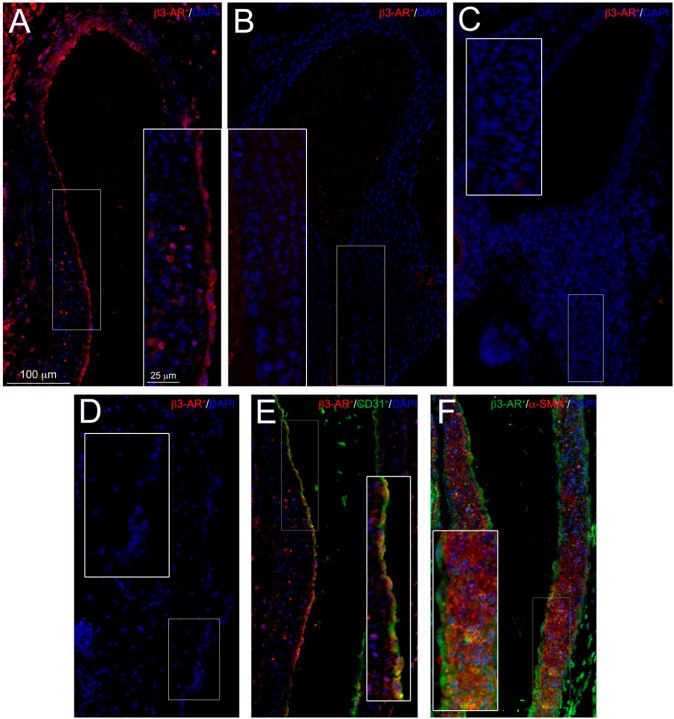
Immunofluorescent staining of β3-AR on the ductus arteriosus (DA). Panel (**A**) β3-AR (red) labeling on DA of prenatal fetuses; Panels (**B**,**C**) β3-AR (red) labeling on DA of newborn mice 1 h and 7 h after delivery, respectively; Panel (**D**) β3-AR (red) labeling is absent on epidermis (negative control); Panel (**E**,**F**) CD31 (green)/β3-AR (red) and α-SMA (red)/β3-AR (green) double labeling, respectively, on DA of prenatal fetuses; CD31/β3-AR and α-SMA/β3-AR positive cells can be seen in the tunica intima and tunica media of the DA. Nuclei are blue stained with DAPI. Scale bars are indicated in panel A. Results are representative of three independent experiments.

**Figure 4:**
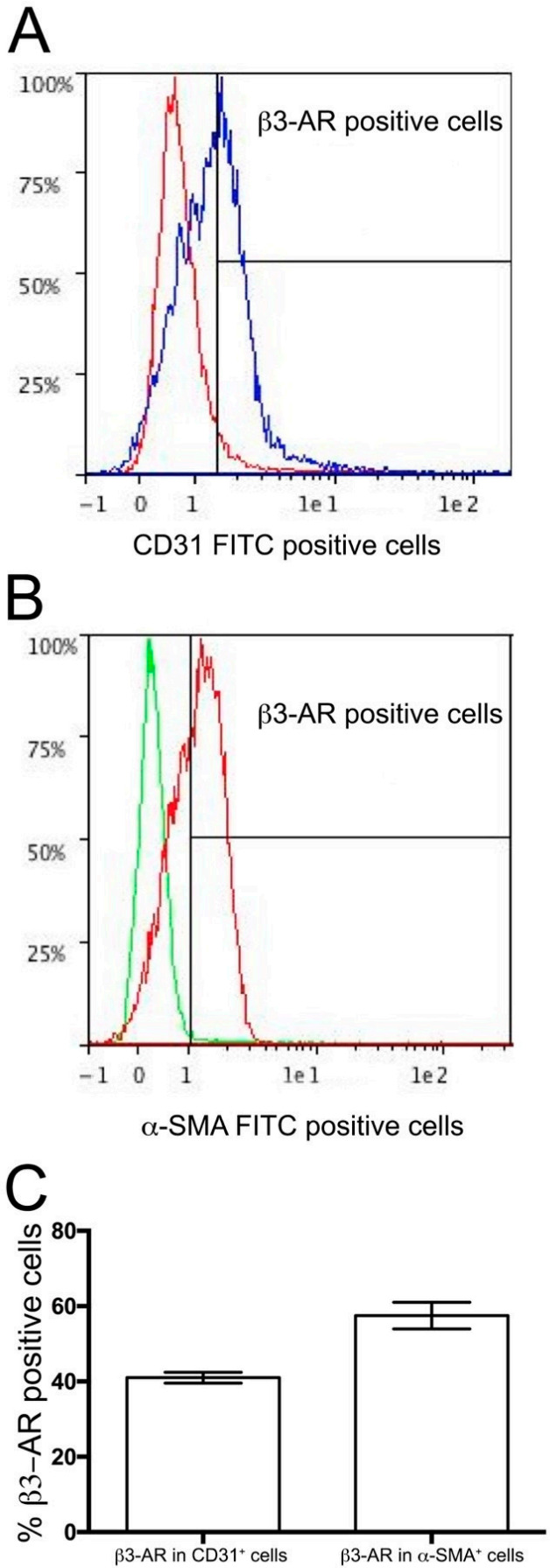
Flow cytometry analysis of β3-AR on the ductus arteriosus (DA). Cytofluorimetric plot of β3-AR positive cells in CD31 positive cells (blue plot) (Panel **A**) and β3-AR positive cells in α-SMA positive cells (red plot). (Panel **B**). About 40% of endothelial cells and 55% of smooth muscle cells are positive for β3-AR (Panel **C**). Results were reported as mean ± SD and are representative of three independent experiments.

The expression of the β3-ARs on DA was evaluated at both mRNA and immunoreactive protein level. Real time RT-PCR clearly demonstrated the presence of receptor transcript on fetal DA at level comparable to that of the bladder, assumed as positive control (Figure 2), whereas no signal was detected on DA of newborns 7 h after delivery. Immunofluorescent evaluation performed on fetal and post-natal DA confirmed the presence of β3-ARs on DA during intra-uterine life (Figure 3A), whereas weak or no signal of this receptor was detected on DA harvested from newborns 1 h after delivery (Figure 3, panel B). Seven hours after delivery, no differences in β3-ARs expression were revealed between DA and epidermis (Figure 3C,D), assumed as negative control. Immunofluorescent and flow cytometry analysis also allowed us to identify the cell types of fetal DA expressing β3-AR. As shown in Figure 3, this receptor was expressed by both endothelial cells (Figure 3E) and smooth muscle cells (Figure 3F). This result was confirmed by flow cytometry analysis, which revealed that about 40% of endothelial cells (Figure 4A,C) and 55% of smooth muscle cells (Figure 4B,C) were positive for β3-AR staining cells.

### 3.3. Response of the Fetal DA to Acute SR59230A Exposure

**Figure 5:**
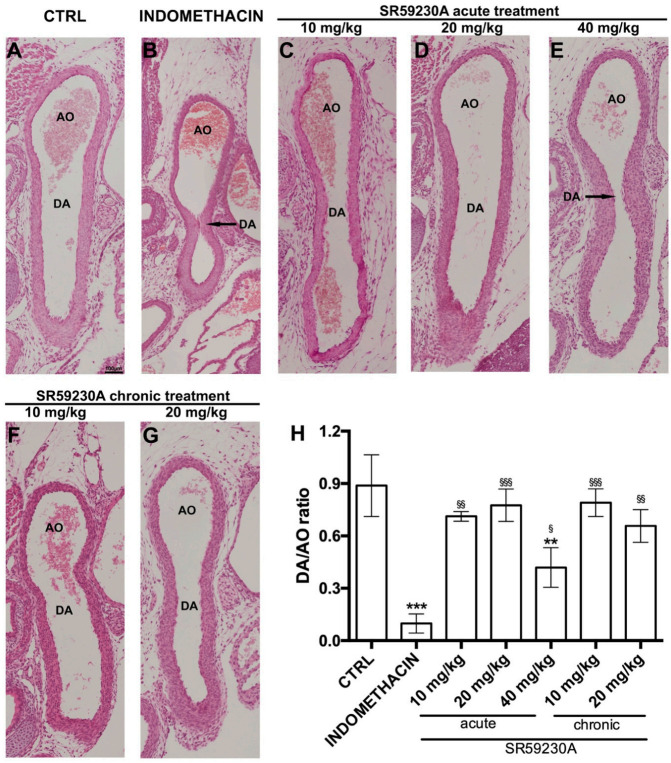
Morphometrical analysis of the ductus arteriosus (DA) patency. Representatives pictures of H&E-stained DA histological sections (panels **A**–**G**) and morphometrical analysis of mean DA diameter, expressed a DA/AO ratio (panel **H**) from fetuses of each experimental group. Compared with the untreated animals (CTRL), acute administration of 40 mg/kg SR59230A or 20 mg/kg indomethacin caused significant reduction of DA patency. AO = descending Aorta. Scale bars are indicated in each panel. At least 20 fetuses of 5 litters were evaluated for each experimental group. Values are means ± S.E.M. and are representative of five independent experiments. (one-way anova test), ^§^ *p* < 0.05, ^§§^ *p* < 0.01 and ^§§§^ *p* < 0.001 versus indomethacin; ** *p* < 0.01 and *** *p* < 0.001 versus control.

To assess the effects of acute administration of the β3-AR antagonist SR59230A on DA patency, pregnant dams were given a single injection of this compound at 10, 20, or 40 mg/kg 6 h before the delivery of pups by cesarean section (protocol A). The results were compared with those obtained administering a single dose of 20 mg/kg indomethacin (protocol B), which is known to cause marked DA constriction [33]. Morphometrical analysis of DA lumen diameter was carried out on hematoxylin/eosin stained sections. The acute treatment with SR59230A did not induce a reduction in the DA lumen size (Figure 5C,D,H) of fetuses at the lowest 10 or 20 mg/kg doses compared with controls (Figure 5A), whereas it induced severe constriction and increased wall thickness of DA at the highest 40 mg/kg dose (Figure 5E,H). As expected, DA was markedly constricted in fetuses following a single administration of indomethacin (Figure 5B,H), which induced a significantly greater caliber reduction than SR 59230A (Figure 5H).

**Figure 6:**
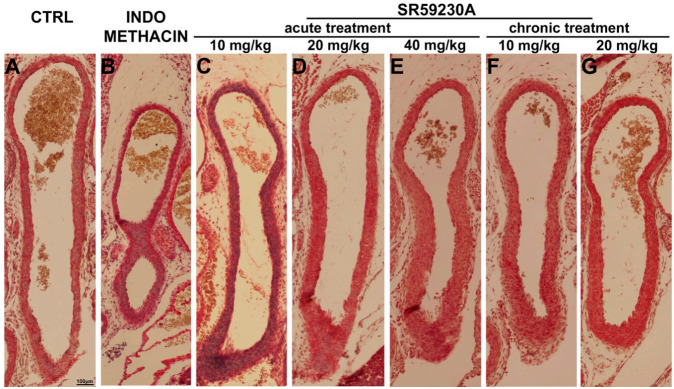
Morphological evaluation of the ductus arteriosus (DA) wall. Representatives pictures of Mallory’s trichrome stained sections of DA from fetuses of each experimental group (**A**–**G**). Compared with the CTRL, untreated animals, (**A**), both the treatment with 40 mg/kg SR59230A (**E**) or 5 mg/kg indomethacin (**B**) increased the overall thickness of SMC layers in the tunica media without affecting collagen content. The treatment with SR59230A at 10 or 20 mg/kg did not alter the DA wall (**C**,**D**,**F**,**G**). Scale bar is indicated in panel **A**. At least 20 fetuses of 5 litters were evaluated for each experimental group and the results reported are representative of five independent experiments.

To investigate whether the effect of SR59230A on DA patency could be induced by vascular wall remodeling, Mallory’s trichrome and paraldehyde fuchsin staining was performed. The morphologic characteristics of DA wall in the animals treated with SR59230A at 10 or 20 mg/kg are comparable to those observed in the controls: in particular the tunica media consisted of elastic lamellae alternating with layers of SMCs, and no detectable accumulation of collagen fibers in the extracellular matrix (ECM) in the subendothelial region of the tunica intima (Figure 6A,C,D). Both SR59230A (40 mg/kg) and indomethacin (20 mg/kg) appeared to increase the number of SMC layers without affecting collagen fiber distribution (Figure 6C,E).

**Figure 7:**
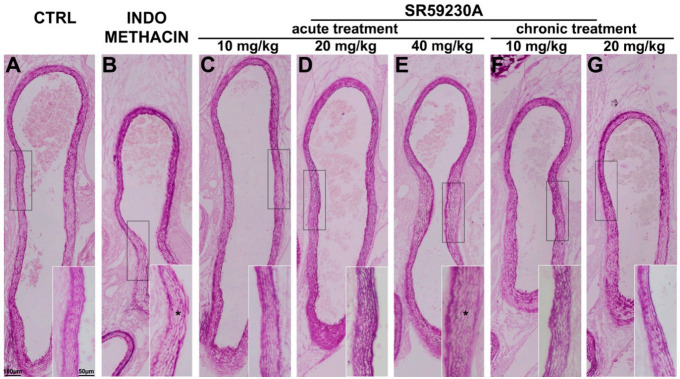
Morphological evaluation of the elastic fibers of the ductus arteriosus (DA) wall. Representative pictures of paraldehyde fuchsin stained sections of DA from fetuses of each experimental group (**A**–**G**). Compared with the untreated mice (CTRL) (**A**), the animals given 40 mg/kg SR59230A (E) or 20 mg/kg indomethacin (**B**) exhibited disassembled, sparse and thin elastic fibers in tunica media (asterisks). The treatment with SR59230A at 10 or 20 mg/kg did not alter the elastic fibers of the ductal wall (**C**,**D**,**F**,**G**). Scale bar is indicated in panel **A**. At least 20 fetuses of 5 litters were evaluated for each experimental group and the results reported are representative of five independent experiments.

The morphological evaluations carried out on paraldehyde fuchsin staining did not reveal significant differences in the elastic fibers of the ductal wall of the animals treated with SR59230A at 10 mg/kg or 20 mg/kg compared with the controls (Figure 7A,C,D). On the contrary, the animals which received SR59230A at the highest dose or indomethacin exhibited sparse, disassembled, and thinner elastic fibers in tunica media (Figure 7A,E), which are typical hallmarks of DA vascular remodeling.

### 3.4. Response of the Fetal DA to Chronic SR59230A Exposure

To examine the effects caused by prolonged exposure of DA to the β3-AR antagonist, pregnant females were daily treated with SR59230A at 10, 20 or 40 mg/kg from day 15.5 to 18.5 of gestation; caesarian deliveries were carried out at day 18.5 of gestation 6 h after the last administered dose (protocol D). The treatment with SR59230A at 10 or 20 mg/kg did not affect the patency of fetal DA (Figure 5F–H) and the histological evaluation carried out on sections stained with Mallory’s trichrome and paraldehyde fuchsin did not reveal signs of vascular remodeling (Figure 6 and Figure 7F–G). On the contrary, SR59230A administration at the highest dose (40 mg/kg) caused preterm delivery in all the animals tested and consequent fetal death, making DA measurements impossible.

### 3.5. Newborn DA Closure after SR59230A Exposure In Utero

**Figure 8:**
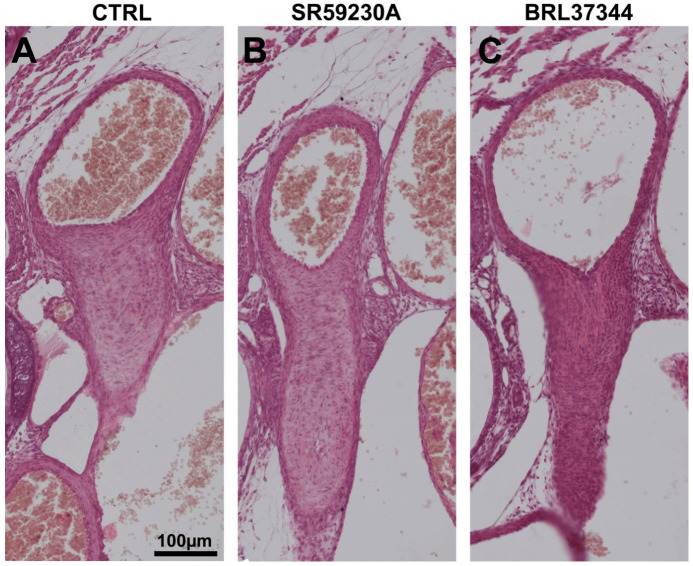
Histological analysis of the ductus arteriosus (DA) in newborn mice. Representatives pictures of DA from pups 7 h after delivery. The DA was completely closed in all the animals of CTRL group (untreated animals), SR59230A (**B**) and BRL37344 (**C**) treated. Scale bar is indicated in panel **A**. Results are representative of three independent experiments.

To exclude that maternal SR59230A administration during pregnancy could impair the ability of the newborn DA to constrict after birth, pregnant females were treated with SR59230A in acute and in chronic mode and pregnancies were allowed to continue until term (protocols C and E). Morphological analysis performed on the tissues harvested from newborn pups 7 h after delivery clearly demonstrated complete DA closure in all the animals (Figure 8A,B), indicating that fetal SR59230A exposure did not interfere with ductal closure after birth.

### 3.6. Response of the Neonatal DA to BRL37344 Exposure

The possible involvement of β3-AR targeting in postnatal DA closure was also examined in experiments whereby a specific receptor agonist BRL37344 was administered to newborn pups within 2 min after birth. Tissues were harvested after 1, 2, and 7 h after treatment and subjected to morphological evaluations. After 7 h following delivery, DA were closed in all the BRL37344 treated animals and showed histologic findings comparable to control mice (Figure 8A,C). This finding excludes that β3-AR stimulation could interfere with postnatal DA closure.

## 4. Discussion

This in vivo study first shows pre- and post-natal effects caused by the exposure of DA to the β3-AR antagonist SR59230A during pregnancy. There has been limited research on β3-AR involvement in morphogenesis, with the only available studies reporting weak umbilical vessel vasodilatation [24] and an increase of fetal adipocyte thermogenesis exerted by its activation [25]. Meanwhile, the consequences of β3-AR targeting, in particular its blockade, for the fetus remains an unexplored field, in spite of the fact that this receptor is highly expressed during the first week of pregnancy [34], probably as a consequence of the relatively hypoxic fetal environment.

A first significant finding of the present study is that β3-AR is expressed by both endothelial and smooth muscle cells of fetal DA, suggesting its possible involvement in maintenance of DA patency. During intra-uterine life, together with high level of PGE_2_ [28], the exposure to low pO_2_ guarantees DA patency [29] and high levels of β3-AR expression. Immediately after birth, coincidentally with the physiological increase in arterial oxygen tension, DA, which is particularly sensitive to oxygen, constricts and finally closes in a few hours [28]. Although a direct correlation between increased level of oxygen and β3-AR expression reduction is not has been established, our findings suggest that the change in oxygen tension occurring after delivery could also decrease β3-AR expression and that, in turn, may contribute to DA constriction. This hypothesis could also explain the ineffectiveness of the β3-AR specific agonist, BRL37344, administered immediately after birth, in maintaining DA patency. Clearly, this hypothesis warrants further investigation.

A second major finding of this study is that SR59230A is able to readily cross the placental barrier and enter the fetal circulation at high concentration. In fact, the fetal blood contained 68.66 ± 2.47% of the SR59230A present in maternal blood: this finding justifies a more detailed investigation into the possible fetal side effects of this compound necessary to propose β3-AR as a therapeutic target of diseases during pregnancy. In this respect, it has recently been demonstrated that β3-AR is widely expressed in the most frequent neoplasms occurring in pregnant women [22] and its blockade reduces cancer progression [14,15,16,17,18]. In particular, SR59230A, administered intratumorally at 5 mg/kg, has been shown to decrease melanoma growth by inducing neoplastic cell death and inhibiting tumor vascularization [15] while, when administered intraperitoneally at 10 mg/kg and 20 mg/kg, it reduced neuroblastoma [18] and melanoma [35] progression, respectively. These latter studies, indicating that SR59230A exerted marked anticancer effects upon intraperitoneal administration at the noted dosage [35], were the background to the present experiments in which we investigated the effects on the DA of SR59230A, administered intraperitoneally at 10, 20 and 40 mg/kg, both in acute and chronic mode. Of note, this study was carried out on healthy dams, without evaluating the anticancer properties of SR59230A. Additional studies are eventually required to demonstrated that this compound is able to maintain its anticancer effect also during pregnancy

The third and most important finding of the present study is that SR59230A, administered at the same doses effective as anticancer therapy (10 and 20 mg/kg) in both acute and chronic mode, neither induced fetal DA constriction nor impaired DA closure after birth. Indeed, the morphologic characteristics of DA wall in the animals treated with SR59230A at 10 or 20 mg/kg were similar to those observed in the controls, excluding any SR59230A-induced DA remodeling. These data in pregnant mice represent preliminary information concerning the safety profile of SR59230A, although additional studies are undoubtedly required. In contrast, SR59230A administered at 40 mg/kg in acute or chronic mode did cause DA restriction and preterm delivery, respectively. The results obtained by the administration of SR59230A at 40 mg/kg in acute mode were compared with those obtained with a single dose of indomethacin, a cyclooxygenase (COX) inhibitor known to cause fetal DA constriction by decreasing PGE_2_ level [32,36,37]. Albeit indomethacin induced a significantly greater caliber reduction of DA caliber than SR 59230A, both compounds were able to promote not only smooth muscle cell contraction, typical features of DA functional closure [27], but also changes in the arrangement of the elastic fibers, which appeared sparse, disassembled, and thin. This alteration of the elastic fibers, although not accompanied by an accumulation of collagen fiber in the subendothelial tunica intima, represents a hallmark of vascular remodeling, that eventually contributes to permanent DA closure [38,39].

A final point deserving comment concerns preterm delivery and consequent fetal death induced by chronic administration of SR59230A at the highest dose (40 mg/kg). Since β3-AR promotes dilatation of umbilical vessel [24], it is possible that a high dose of this β3-AR antagonist may have induced their vasoconstriction, causing severe fetal ischemia and death [40]. Another possible explanation relies on the observation that β3-AR is upregulated in the pregnant myometrium where it inhibits spontaneous contractions [4,41]. Hence, SR59230A at high dose may have interfered with the tocolytic effect of β3-AR, inducing preterm delivery. It has been also reported that SR59230A at a high dose could act as an α1-adrenoceptor antagonist [42], thus possibly interfering with orthosympathetic-induced myometrial contractility [43]. These hypotheses are not mutually exclusive and provide background to further pharmacological investigations on SR59230A to better elucidate its effects and underlying mechanisms of action. In addition to the above points, a recent study has shown that β3-AR is involved in the mechanisms of fetal immune tolerance [44]. Hence, it cannot be ruled out that β3-AR blockade by SR59230A may have increased the occurrence of abortion through an immune-mediated mechanism, although this is unlikely because of the very late stage of pregnancy at the time of drug delivery.

In conclusion, SR59230A, administered at the doses effective in reducing cancer progression (10 and 20 mg/kg) in both acute and chronic mode, did not induce fetal DA constriction. Although we are well aware that the present findings from an animal model cannot be directly transferred to clinical practice, we deem that they do suggest that β3-AR antagonists may be a new therapeutic approach to cancers in pregnant women. On the other hand, our data also suggest that caution is mandatory if planning to administer SR59230A at high doses at term gestation, because of the high risk of fetal adverse effects due to DA constriction.

## Figures and Tables

**Table 1:** List of primary and secondary antibodies.

Primary Antibodies	Dilution	Producer
β3-AR	1:20/1:100	ab94506; Abcam, Cambridge, UK
CD31	1:20/1:100	ab9498; Abcam, Cambridge, UK
α-SMA	1:500	ab21027; Abcam, Cambridge, UK
α-SMA	1:50	ab7817; Abcam, Cambridge, UK
**Secondary Antibodies**	**Dilution**	**Producer**
Alexa Fluor Anti-rabbit	1:333	Jackson Immuno Reasearch Labs, West Grove, PA, USA
Alexa Fluor Anti-mouse	1:333	Jackson Immuno Reasearch Labs, West Grove, PA, USA
Alexa Fluor Anti-goat	1:333	Jackson Immuno Reasearch Labs, West Grove, PA, USA

**Table 2:** SR59230A blood concentrations.

Treatment	Dam (μg/L)	Fetus (μg/L)
Acute 40 mg/kg	13.1 ± 2.66	9.03 ± 2.01
Acute 20 mg/kg	4.88 ± 0.71	3.49 ± 0.53
Acute 10 mg/kg	2.5 ± 0.49	1.62 ± 0.29
Chronic 20 mg/kg	6.59 ± 0.88	4.47 ± 0.5
Chronic 10 mg/kg	3.51 ± 0.59	2.45 ± 0.33
